# The Cornea in Health and Disease

**Published:** 1875-09

**Authors:** F. W. Abbott


BUFFALO

### Medical and Surgical Journal.

VOL XV. SEPTEMBER, 1875. No. 2.

### Original Communications.

:o:

ART. I—*The Cornea in Health and Disease.* By F. W. Abbott, M. D. Read at the Buffalo Medical Association, Sept. 7, 1875.

We read, in ancient story, of two valiant knights who approached a suspended shield from opposite sides, and as one said that it was of silver, while the other knew that it was golden, their knightly honor considered this question of veracity capable of no other solution than the appeal to arms. When both lay prostrate on the field with life ebbing, the shield was reversed, and each saw that the other was right, from his point of view.

Many a battle long and fierce has been fought since that day, by contestants too eager for frays to essay a look at the other side of the shield before spurring upon their adversaries.

If we seem, in considering our subject to-night, to ignore the views of some of our friends who always stand upon evolutionary ground, let them not say that in ascribing an exquisite adaptation of means to an end to a beneficent creator, we enter the lists against any one. It is only that our attention is so absorbed by the perfections of the golden side, that we have no place left for thought or care for any baser obverse.

Presupposing the existence of the other parts of the eye, its nerve, refracting media, and tunics, let us look for a moment at some of the indications to be fulfilled in supplying its anterior segment; and then consider how these indications are met.

It is evident, in the first place, that it must be perfectly transparent, to allow the rays of light to pass unimpeded to the sensitive retina; and the index of refraction of all its component parts must be the same, that these rays be not distorted or turned out of their way. It must be of sufficient consistence to preserve its shape perfectly, in spite of the intraocular pressure, or any ordinary force which may act upon it externally. Its nutrition must be so provided for that it may heal rapidly after an injury, and resist the onset of disease. And it must be kept perfectly clean, lest any adhering impurity impair its perfect transparency. None of the tissues of the body as they appear in other places, fulfill these conditions, but they are met so completely by an adaptation of tissues and processes occuring elsewhere, that we gladly recognize a Supreme hand working in the whole body with unity of purpose for the needs and happiness of its immortal occupant.

In examining the structure of the cornea, we find that it is composed broadly of four layers: 1st. The external epithelium. 2d. The true corneal tissue. 3d. The membrane of Descemet. 4th. The internal epithelium.

The external epithelium is laminated, flat epithelium, in the adult 0.03 mm. in thickness.

The membrane of Descemet is a transparent, structureless membrane, 0.006–0.008 mm. thick in the center, and 0.01–0.012 mm. at the border.

The internal epithelial layer is a simple layer of flattened cells lying on the membrane of Descemet, called the endothelium of the membrane of Descemet. These three layers present no points of particular interest.

The true corneal layer is about 1 mm. in thickness at the edges, and a little thinner in the center. It is built up of lamellae, which lie parallel with its surface, and are composed of broad thin layers of very delicate fibrillae. Between these lamellae are interspaces, which anastomose with each other and have received different names, according to the varying views of their nature entertained by different anatomists. They consist of flat, nucleated cells, without a cell membrane, lying for the most part parallel with the surface of the cornea. These corneal corpuscles have numerous processes which pass off from them in every direction, and unite to form a cellular network, traversing the entire cornea.

By this arrangement of the corneal elements, we see how perfectly the indications as to consistence and nutrition are fulfilled. The tough connective tissue fibrillae giving sufficient firmness, and the cellular network affording a supply of nutrition to every part. The transparency and optical homogeneity are provided for by making the fibrillae transparent, and glueing them together by a cement, and filling the corpuscles with a protoplasmic substance which possess the same index of refraction as the true corneal tissue This cellular network is far too fine in health to permit the passage of the blood globules, but in disease we see the delicate tubes rapidly enlarging until, as practical blood vessels, they carry the rich red flood to battle against the corroding ulcer which threatens destruction, and fill it up again with firm, unyielding tissue.

The sensitiveness of the cornea is ensured by a plexus of nerve fibres which lose their medullary sheath near its margin, and so penetrate its substance without rendering it opaque. When any speck alights upon its surface these alert sentinels send the alarm to lid and lachrymal gland, who with brush and water so quickly sweep away the intruder that the tenacious retina, holding its impression for an instant, is entirely unconscious of any obstruction to its vision.

Notwithstanding all these means so beneficently applied for its nutrition and preservation, the cornea is not exempt from the ills and accidents of other organisms, and we will look for a few minutes at some of these, and the means for their relief.

The transparence of the cornea renders its disorders peculiarly interesting to the observer, for here he may see the rise and progress of disease which elsewhere is hidden from sight, and he may recognize at once conditions which in other places would be revealed only by results. This exposure to observation is doubly precious here, where the loss of a few atoms of tissue more or less, which anywhere else would be a matter of no moment, may make the difference between a life of hopeless darkness and the priceless blessing of sight.

Perhaps the most common of the uncomplicated affections of the cornea is Phlyctenular keratitis or Herpes corneae. This appears as a circumscribed, rounded, cloudy nodule or point of inflammation in the anterior layers of the cornea, with the subjective symptoms of lachrymation, photophobia, and ciliary neuralgia. This nodule may pass away by resolution and leave no sign, or it may be surmounted by a vesicle which quickly bursts, or the superficial layers may be thrown off, in either case leaving an ulcer.

The treatment of this affection is very simple when seen in its incipiency. The insufflation of a little levigated calomel, with the use of a mild solution of atropia usually suffices to affect a cure in a few days. Strong astringent or irritating applications are decidedly contra-indicated in this trouble, especially any form of nitrate of silver, which often prevents the resolution which we wish to obtain and changes the phlyctenula into an ulcer, of which we shall soon speak.

As I said one phlyctenula is easily treated, but how to prevent others from coming, and coming again and again, "Ay, there's the rub." I have found the exhibition of quinine to be more efficacious in breaking up this habit of recurrence than any other drug, but this must be aided by open air exercise, or a change of air, brine baths, excluding pork and pastry from the bill of fare, etc. This disease, called by some strumous ophthalmia, has not, in my hands, been at all benefitted by iodide of potassium and similar anti strumous remedies. In obstinate cases of recurring phlyctenulæ where the lids tightly hug the eye-ball, recourse may be had to the operation of canthoplasty as recommended by Dr. Agnew in a recent article on "Canthoplasty as a remedy in certain diseases of the eye."

Ulcers of the cornea, from their frequent occurrence, come under the observation of every physician, and as their results are often lamentable, they claim a prominent place among corneal troubles. These are of all grades of severity. Sometimes they appear as minute excavations which escape the patients notice, the photophobia and lachrymation being ascribed to a "cold in the eye"

until, the danger and suffering being at an end, the minute nebula of new tissue brings him, in great alarm, to the physician on account of a film which is growing over the sight. Sometimes we see the broad perforating ulcer, which leaves in its train a bulging staphyloma of the iris, which causes hideous deformity, blindness, and even the loss of the whole eyeball.

This is not the occasion for a detailed description of the manifold phases of corneal ulceration. I will only speak of some of the means at our command for their relief, and the amelioration of some resulting deformities.

The rationale of the treatment in an ulcer on the cornea differs from that for treating a similar condition in any other part of the body. Elsewhere we try to heal an ulcer as quickly as possible; here we are concerned not only with its healing, but how it heals, for we find that if the new tissue is deposited slowly it is much more likely to be transparent than when it fills up rapidly. So we try to check the ulcerative process as soon as possible, and then to guide the nutrition so that the edification may be slow and gradual, but constant.

In small ulcers unaccompanied by much irritation a gentle stimulant is required, and for this I employ the insufflation of calomel more often than any other remedy. This causes so little pain that the most sensitive child seldom resists its second application. It should not be employed, however, together with the internal use of pot. iodidi, as they occasionally form a very irritating compound in the eye. A collyrium of atropia sulph. is indicated in almost every case, to soothe the exposed nerve fibres, and by its action in decreasing the intraocular pressure to relieve the strain upon the weakened cornea, and thus lessen the danger of perforation. One caution should be observed in the use of atropia. Some druggists keep only the simple alkaloid, which is insoluble in pure water, and they make a solution by adding an acid which may prove irritating instead of soothing. The neutral sulphate which dissolves perfectly in water is always preferable. If the subjective symptoms of photophobia and ciliary neuralgia are intense, much benefit is often derived from painting the brow with tr. iodine, in which morphine has been dissolved in the proportion of from 10 to 20 grs. to the ounce. Some years ago I learned from Dr. Rochester that the excessive photophobia which we sometimes meet with in this disease, may often be so relieved by the inhalation of chloroform, that after the anesthesia has passed off the eye remains much less sensitive to light than before.

Here also the practice of touching the ulcer with a pointed stick of nitrate of silver, or a brush dipped in a strong solution of this salt is not only cruel, on account of the useless pain which it inflicts, but often detrimental in destroying the thin protecting layer of epithelium which, in the regeneration of the lost tissue, is thrown over the walls and floor of the cavity. Another remedy to be avoided is acetate of lead, which often leaves an ineradicable white deposit at the site of the ulcer.

When an ulcer does not yield to mild remedies a paracentesis of the cornea should be performed without delay. This is easily done by using a broad needle and puncturing the cornea near its border. Upon slightly rotating it the aqueous humor escapes, and although it collects again in a few hours, the temporary relief from strain often permits the floor of the ulcer to gain sufficient strength to resist the pressure when the aqueous collects again. This little operation is unfortunately contra-indicated in cases complicated with blennorrhea of the lids, as then the edges of the wound may become infected.

In sluggish, sloughing ulcers, recourse may be had to hot compresses, to promote the flow of blood to the part, but their use is attended by so much danger that they never should be left to the nurse, but should be applied and the effect watched by the attending surgeon. When there is much conjunctival inflammation and the nutritition of the cornea is strangulated by the swelling, cold applications may be made, but these, too, must be carefully watched, lest they decrease too much the vitality of the already enfeebled cornea.

In spite of all our care a corneal ulcer sometimes heals only at the expense of a leucoma of greater or less density, and these, especially when central, interfere so much with the vision and cause such deformity that their treatment becomes a matter of great importance. In leucomata which are not very large and dense the probabilities are that they are composed of true corneal tissue, but so irregularly deposited as not to be transparent. In these cases the object of treatment should be to keep up such a degree of irritation in the eye as will promote tissue change without causing inflammation. To accomplish this it is better to begin with mild applications, as calomel, and proceed to more irritating ones, as quinine, the amorphous yellow suboxide of mercury in some mild unguent, etc. This last preparation I have found of much service in such cases, and commonly use cosmoline as a vehicle, beginning with ℞ Hydrarg subox. flav. grs. iii, cosmoline ʒii., increasing the strength *pro re nata.* Of this a portion as large as a grain of rice should be applied every other day.

As this preparation of mercury is not officinal, although mentioned in the last Dispensatory, a little care must be taken in prescribing it. A very competent druggist, who never had happened to see it, substituted turpeth mineral for it in one of my prescriptions a while ago, and the consequences were not pleasant.

A few years ago, in a memoir to the French Academy, M. De Luca gave glowing accounts of the effect of finely powdered suphate of soda in dissolving corneal opacities, but I think that, in the hands of American practitioners at least, it has been found to be of no more service than other applications.

Sometimes, unfortunately, we see as the result of an ulcer a dense, white cicatrix covering the pupil, and perhaps occupying the greater part of the cornea, as imperishable as any other scar, causing total loss of sight and hideous deformity. When there is any clear cornea the well known operation of iridectomy may restore a good degree of sight.

To relieve the deformity caused by the covering of the black pupil with a dense white cicetrix, the device of tattooing the cornea has lately been followed, and the cosmetic effect of this operation is sometimes very happy. Dr. Scott, of Cleveland, told me of a case, a few days since, upon which he operated, where the patient, a young man, found it impossible to get a situation, his appearance was so repulsive. The operation was a complete success, and a few months afterward he called upon the Doctor and said that the operation had been worth hnndreds of dollars to him, as now he could get a situation without difficulty. Patients frequently come to us to have a "pearl" taken off from the eye, who care scarcely anything about the loss of sight in one eye, as they suffer very little inconvenience from that, but they are very much concerned about their appearance, and will undergo much to be relieved from this blemish. The idea of the operation is very simple. It consists in transforming a dense glistening white surface into a black one by pricking in India ink. Several forms of needles have been constructed for this purpose, one of the most ingenious of which was devised by my friend Dr. Howe, but as it is not yet in the market I have never used it. I use Agnew's instrument, which consists of five fine needles arranged in a row, the line of whose points forms a concave arc corresponding with the convexity of the cornea.

Each surgeon differs from his fellows in the details of this operation, and one cannot treat all cases alike, so as a specimen I will tell you how the patient whom I show you this evening was operated on.

As she has very good courage I did not give chloroform. The tears will start, although the corneal cicatrix is not as sensitive as the normal cornea, so I had her sit upright in order that the tears might flow out below the scar and not dilute the ink. Standing behind the patient, I held her head firmly against the high chair-back, with my left hand on her forehead, and lifted the upper lid with the index. As the eye naturally rolls up when the cornea is touched this left the leucoma exposed without holding the lower lid. I then dipped the needle into a thick paste of India ink, and with it thus charged made a few punctures through the epithelial layer into the substance of the cornea. After holding the lid a moment to give the ink time to settle into the punctnres I let it fall and allowed the eye to rest a few minutes. This process was repeated a few times and the patient dismissed, with directions to use water dressings if much irritation should ensue. This patient has had four sittings, and as after the last one, which was at five o'clock Saturday afternoon, she prepared supper for a family of 18 persons and has done her work every day since, you can judge the amount of inflammation which the operation sets up. Some surgeons prefer to perform the whole operation at one sitting, but I think the ink is more likely to wash out when the whole surface is lacerated at once, and prefer to take even half a dozen sittings since the irritation following is less than that caused by a smart application of the time-honored blue-stone. This operation should not be performed until some months have elapsed since the healing of the ulcer, and the cicatrix has become dense and firm. If any irritability remains, and the eye easily becomes sensitive and inflamed, or if the cicatrix is weak and thin, the operation should be delayed until these conditions are relieved.

There are several other affections of the cornea which, by their importance, seem to demand some attention, but I fear I have already wearied you, and will close by bringing in two or three patients to illustrate some of the points taken.

